# Association of Polymorphisms in miRNA Processing Genes With Type 2 Diabetes Mellitus and Its Vascular Complications in a Southern Chinese Population

**DOI:** 10.3389/fendo.2019.00461

**Published:** 2019-07-12

**Authors:** Zihao Wen, Xiaoqian Zou, Xin Xie, Shaoling Zheng, Xiaojing Chen, Kehui Zhu, Shirui Dong, Jiayu Liang, Xiuxia Huang, Dandan Liu, Yao Wang, Yumei Liu, Jing Wu, Yuting Ying, Kailiang Liu, Congying Lu, Baohuan Zhang, Guang Yang, Chunxia Jing, Lihong Nie

**Affiliations:** ^1^Department of Epidemiology, School of Medicine, Jinan University, Guangzhou, China; ^2^Department of Pathogen Biology, School of Medicine, Jinan University, Guangzhou, China; ^3^Guangdong Key Laboratory of Environmental Pollution and Health, Jinan University, Guangzhou, China; ^4^Department of Endocrine, The First Affiliated Hospital of Jinan University, Guangzhou, China

**Keywords:** T2DM, vascular complication, miRNA processing gene, polymorphism, interaction, RAN gene, DICER1 gene

## Abstract

**Objective:** To evaluate the potential association between the genetic variants in miRNA processing genes (*RAN, XPO5, DICER1*, and *TARBP2*) and susceptibility to type 2 diabetes mellitus (T2DM) and its vascular complications, as well as to further investigate their interaction with environmental factors in type 2 diabetes.

**Methods:** We conducted a case-control study in genotyping of five polymorphic loci, including *RAN* rs14035, *XPO5* rs11077, *DICER1* rs13078, *DICER1* rs3742330, and *TARBP2* rs784567, in miRNA processing genes to explore the risk factors for T2DM and diabetic vascular complications. Haplotype analyses, interactions of gene-gene and interactions of gene-environment were performed too.

**Results:** We identified a 36% decreased risk of developing T2DM in individuals with the minor A allele in *DICER1* rs13078 (OR: 0.64; 95%CI: 0.42–0.95; *P*: 0.026). The AA haplotype in *DICER1* was also associated with a protective effect on T2DM compared with the AT haplotype (OR: 0.63; 95%CI: 0.42–0.94; *P*-value: 0.023). T2DM patients with the TT+TC genotype at *RAN* rs14035 had a 1.89-fold higher risk of developing macrovascular complications than patients with the CC genotype (OR: 1.89; 95%CI: 1.04–3.45; *P*-value: 0.037). We also identified two three-factor interaction models. One is a three-factor [*DICER1* rs13078, body mass index (BMI), and triglyceride (TG)] interaction model for T2DM (OR: 5.93; 95%CI: 1.25–28.26; *P* = 0.025). Another three-factor [*RAN* rs14035, hypertension (HP), and duration of T2DM (DOD)] interaction model was found for macrovascular complications of T2DM (*OR* = 41.60, 95%CI = 11.75–147.35, *P* < 0.001).

**Conclusion:** Our study provides new evidence that two single nucleotide polymorphisms (SNPs) of the miRNA processing genes, *DICER1* and *RAN*, and their interactions with certain environmental factors might contribute to the risk of T2DM and its vascular complications in the southern Chinese population.

## Introduction

Diabetes mellitus (DM) is a chronic disease that occurs when there are increased levels of blood glucose because the body is unable to produce any or enough of the hormone insulin or use insulin effectively (1). According to the International Diabetes Federation (IDF) report, there were 4.25 billion people with diabetes in 2017, and the number of diabetes patients will increase to 6.29 billion globally by 2040 (2). In 2017, more than 4 million people died because of diabetes and diabetic complications, which means that worldwide, a patient died every 8 s because of diabetes (2). The total global healthcare expenditure due to DM was estimated at 727 billion dollars in 2017 (3). As the most populous and largest developing country in the world, China has the highest number of diabetes patients worldwide (114.4 million in 2017) (2). The number of diabetes patients soared from 4.8 million in 1980 to 39.8 million in 2007 (4).

T2DM as the most common type of diabetes, accounts for approximately 90% of diabetes cases and is affected by both environmental factors and genetic factors (5–7). Type 2 diabetes mellitus is a long-term metabolic disorder characterized by damage to insulin secretion and sensitivity, resulting in hyperglycemia (8, 9), which can lead to the development of diabetic vascular complications, such as diabetic nephropathy, peripheral artery disease (PAD) and coronary heart disease (CHD) (2, 10, 11). Risk factors for diabetic vascular complications include lifestyle (such as smoking and drinking), duration of diabetes, age of onset and genetic factors, etc. (12, 13).

**Figure 1:**
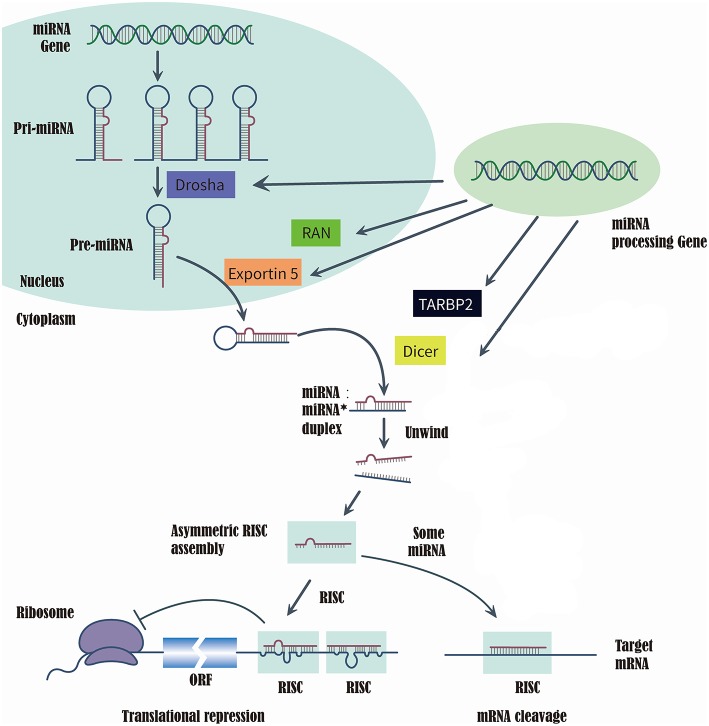
Summarize of the biosynthesis process of miRNA and the function of miRNA processing Gene and enzyme.

MicroRNAs (miRNAs) are a class of short, single-strand, non-coding, and endogenous RNA molecules of 21–23 nts in length (14). Although miRNAs constitute at most 3% of the human genome, it has been reported that approximately one-third of human genes were regulated by miRNAs (15, 16). Recently, the evidences that series of miRNAs were related to the T2DM and diabetic vascular complication, have been proved by several studies (17), such as miR-200 family (18), miR-124a (19), miR-21-5P (20), and miR-125a-3P (20). RAS-related nuclear protein (RAN), exportin 5(XPO5), DICER1, and TARBP2, which are known as microRNA processing enzyme, are the key to complete the biosynthesis of mammalian miRNAs (21, 22). First, RNA II polymerase transcribed miRNAs into long precursors called pri-miRNAs, which are cleaved in the nucleus to release a stem loop intermediate about 60–70 nt, known as the miRNA precursor hairpin (pre-miRNA). Secondly, XPO5 and RAN export the pre-miRNA from the nucleus to the cytoplasm. And the DICER1 and TARBP2 further cooperatively dice the pre-miRNA into a double-stranded, short miRNA duplex (23, 24). Last, the double-stranded miRNA is preferentially incorporated into RNA-induced silencing complex, targeting endogenous mRNA silencing (25, 26). Therefore, if the expression and structure of these miRNA processing proteins have been altered in the biosynthesis of mammalian miRNAs, it would directly impact the biosynthesis of mature miRNAs and further change the function and structure of miRNAs (21, 22) (Figure 1).

A variation in a single nucleotide is called a single nucleotide polymorphism (SNP) that occurs in polymorphisms of the genomic DNA sequence, and is the simplest form of DNA variation among individuals and might affect the expression and function of genes (27). It has been reported that SNPs are associated with the development of type 2 diabetes mellitus and its vascular complications (28–30). GWAS (genome-wide association studies) have identified over 80 SNPs were closely related to type 2 diabetes mellitus (31–34). In addition, GWAS also identified over 20 SNPs related to the various chronic T2DM complications (e.g., diabetic nephropathy, diabetic retinopathy, diabetic cardiopathy and diabetic painful neuropathy) (35). *RAN* rs14035, *DICER1* rs3742330, and *DROSHA* rs10719 are located in the 3′-UTR, which may disturb the function of these miRNA processing proteins by impacting the binding site of their miRNAs, resulting in dysregulation in processing of related gene expression and impact the biosynthesis of related proteins (36–39). We hypotheses that the SNPs in miRNA processing genes may impact the susceptibility to T2DM and its vascular complications by disrupting the structure, binding sites, or processing of miRNA (40, 41). To our knowledge, few studies evaluated the potential association between the risk of T2DM and its vascular complications and specific SNPs in miRNA processing genes. Thus, the aim of our study is investigating the effects of variations in miRNA processing genes, *RAN* (rs14035), *XPO5* (rs11077), *DICER1* (rs3742330 and rs13078), and *TARBP2* (rs784567), as well as their interactions with environmental factors on T2DM and its vascular complications in a Southern Han Chinese population.

## Methods

### Study Subjects

This case-control study included a total of 1,275 unrelated Han Chinese Southern residents in Guangzhou. A total of 743 T2DM subjects were inpatients of the Endocrinology Departments of the First Affiliated Hospital of Jinan University in Guangzhou from September 2011 to January 2018, and all patients were diagnosed using the 2003 American Diabetes Association criteria (42). Patients with impaired glucose tolerance and type 1 diabetes mellitus were excluded from the study. During the same period, a total of 532 healthy control individuals without a family history of diabetes and with normal fasting glucose were also randomly recruited to match T2DM patients by sex and age (±5 years). Before participating in the study, all of the included individuals agreed and signed informed consent, and the study protocol was approved by the Ethics Committee of Medical School at Jinan University. Within the 743 included T2DM patients, 46, 96, 108, and 227 cases were diagnosed with T2DM with other complications, micro-macrovascular complications, macrovascular complications, and microvascular complications, respectively, while 266 cases were categorized into T2DM without any complication. According to the 8th edition IDF Diabetes Atlas (2) and Gregg et al. (43), macrovascular complications included coronary artery disease (CAD), resulting in myocardial infarction or stenocardia, and peripheral artery disease (PAD), leading to diabetic encephalopathy and cerebral infarction, whereas microvascular complications are retinopathy, neuropathy and nephropathy. Micro-macrovascular complications were defined as presenting with both macrovascular microvascular and complications.

### Selection of SNPs

We determined the targeted SNPs of *RAN*/*XPO5*/*DICER1/TARBP2* were through an electronic search of the HapMap database based on the genotype information of Han Chinese in Beijing, China. And, the tarSNPs were determined based on the following criteria: (1) the minor allele frequency (MAF) of SNPs >5%; (2) pairwise tagging with *r*^2^ ≥ 0.8; and (3) *P*-value of Hardy-Weinberg > 0.001. Five SNPs were identified, *RAN* rs14035, *XPO5* rs11077, *DICER1* rs3742330 and rs13078, and *TARBP2* rs784567. Finally, five target SNPs were selected for further analysis. The detailed information of the five tarSNPs, including alleles, MAFs, genes locations, the result of Hardy-Weinberg equilibrium and call rate, is shown in Table S1. However, rs14035 in the *RAN* gene did not meet the Hardy-Weinberg equilibrium (*P* < 0.05). Therefore, the further analyses of the polymorphism associations within T2DM group and health control group excluded rs14035 in the *RAN* gene.

### DNA Extraction and Genotyping

A QIAmp Blood DNA Mini Kit (Qiagen, Hilden, Germany) was used to extract the genomic DNA from peripheral whole blood samples. A Sequenom MassARRAYiPLEX Gold analyzer (Sequenom, Life Technologies, Shanghai) was used to genotype the five selected SNPs. MassARRAY Assay Design 3.1 software were used to design PCR conditions and primers (Table S1).

### Statistical Analysis

The demographic data and clinical characteristics of the included individuals are presented as the mean ± S.D. or number (percentage). The Shapiro-Wilk test was performed to identify the normality of the included demographic data and clinical characteristics in each comparison group (Tables S2–S5). For continuous variables with normal distribution, the *t*-test and ANOVA were performed on them, otherwise the non-parametric test (Kruskal-Wallis test and Mann-Whitney test) was conducted. For categorical variable, the χ^2^-test was performed. The logistic regression with different genetic model (codominant, dominant, and recessive models) was performed to calculate the ORs and 95% confidence intervals (CI) for estimating risk (44). Hardy-Weinberg equilibrium (HWE) of each SNP in the control group was calculated by χ^2^ statistics. All the above statistical analyses were performed by SPSS software v.22.0 (SPSS, Inc.).

The MDR (multifactor dimensionality reduction) software (45) was used to performed to identified the best model of gene-gene interaction models and gene-environment interaction models of the five selected SNPs. The environmental factors we included in MDR were age, gender, BMI (body mass index), TG (triglyceride), TC (total cholesterol), LDL (low density lipoprotein), HDL (high density lipoprotein), FBG (fasting blood glucose), post-prandial blood glucose, HbA1c, duration of T2DM, patients with family history of diabetes, former smoking, current smoking, current smoking, current drinking, and patents with hypertension. SHEsis, an online software (http://analysis.bio-x.cn/myAnalysis.php), was used to identified the effects of haplotype frequencies on T2DM and its vascular complications (46–48).

## Result

### Population Characteristics

The baseline population characteristics of all included individuals are presented in Table 1. In comparison with healthy controls, the patients in T2DM group had higher levels of body mass index (BMI), triglyceride (TG), fasting blood glucose (FBG), GPT (glutamic-pyruvic, transaminase), serum creatinine, and blood uric acid but lower levels of low-density lipoprotein (LDL) and high-density lipoprotein (HDL), when compared with healthy controls. In comparison with T2DM patients without any complications, all T2DM patients with microvascular complications, macrovascular complications and micro-macrovascular complications had higher average age, longer duration of T2DM, and high levels of serum creatinine, but they had lower estimated glomerular filtration rate (eGFR) and GPT levels. Additionally, in comparison with T2DM patients without any complications, hypertension was more prevalent in T2DM patients with vascular complications (macrovascular, microvascular, and micro-macrovascular complications). And T2DM patients with macrovascular complications included a larger number of former smokers (Table 2).

**Table 1:** The baseline population characteristics of healthy controls and T2DM patients.

**Characteristics**	**Healthy controls (*N* = 532)**	**T2DM patients (*N* = 743)**	***P*-value**
Age (years)	61.82 ± 13.05	61.06 ± 13.18	0.385^Δ^
Gender (Male/Female)	251/280	359/384	0.712^Δ^
BMI (kg/m^2^)	21.96 ± 2.60	**24.24** **±** **3.32**	<0.001^**^^Δ^
Triglyceride (mmol/L)	1.35 ± 0.78	**2.10** **±** **1.85**	<0.001^**^^Δ^
Total cholesterol (mmol/L)	5.16 ± 0.88	5.09 ± 3.28	0.576^Δ^
LDL (mmol/L)	**3.08** **±** **0.79**	2.89 ± 1.18	0.001^**^^Δ^
HDL (mmol/L)	**1.51** **±** **0.34**	1.13 ± 0.40	<0.001^**^^Δ^
Fasting blood glucose (mmol/L)	5.25 ± 0.74	**9.40** **±** **4.69**	<0.001^**^^Δ^
Glutamic-pyruvic transaminase (IU/L)	19.87 ± 9.80	**28.22** **±** **36.27**	<0.001^**^^Δ^
Serum creatinine (umol/L)	74.16 ± 16.22	**84.08** **±** **80.12**	0.001^**^^Δ^
Blood uric acid (umol/L)	337.93 ± 85.84	**363.62** **±** **120.46**	<0.001^**^^Δ^
Post-prandial blood glucose (mmol/L)	NA	15.81 ± 5.88	NA
HbA1c (%)	NA	9.03 ± 2.59	NA
Fasting C-peptide (ng/ml)	NA	1.50 ± 1.28	NA
Post-prandial 1 h C-peptide (ng/ml)	NA	2.99 ± 3.04	NA
Post-prandial 2 h C-peptide (ng/ml)	NA	3.84 ± 3.99	NA
eGFR (mL/min)	NA	76.22 ± 21.22	NA
Duration of diabetes (years)	0	7.74 ± 6.65	NA
Patients with family history of diabetes [*n* (%)]	NA	157 (21.13%)	NA
Former smoking [*n* (%)]	NA	31 (4.17%)	NA
Current smoking [*n* (%)]	NA	124 (16.70%)	NA
Current drinking [*n* (%)]	NA	64 (8.63%)	NA
Patients with Hypertension [*n* (%)]	NA	271 (36.47%)	NA

** *P < 0.001*.

Δ *the P-value of χ^2^-test; the P-values of Mann-Whitney test*.

*BMI, body mass index; LDL, low density lipoprotein; HDL, high density lipoprotein; HbA1c, glycated hemoglobin*.

*The bold in the table means statistically significant (P <0.05)*.

**Table 2:** The baseline population characteristics of T2DM subgroups.

**Characteristics**	**T2DM without complication (*n* = 266)**	**T2DM with microvascular complications^a^ (*n* = 227)**	***P*-value**	**T2DM with macrovascular complications^a^ (*n* = 108)**	***P*-value**	**T2DM with microvascular-macrovascular complications^a^ (*n* = 96)**	***P*-value**
Age (years)	56.73 ± 13.15	**60.57** **±** **12.59**	<0.007^**^^∧^	**66.98** **±** **10.76**	<0.001^**^^∧^	**68.62** **±** **10.94**	<0.001^**^^∧^
Gender (Male/Female)	125/141	104/123	0.798^Δ^	60 / 48	0.133^Δ^	42 / 54	0.585^Δ^
BMI (kg/m^2^)	24.26 ± 3.34	24.23 ± 3.33	0.920^∧^	24.39 ± 3.27	0.746^∧^	24.45 ± 3.23	0.666^∧^
Triglyceride (mmol/L)	2.27 ± 2.25	2.07 ± 1.71	0.657^∧^	1.92 ± 1.23	0.168^∧^	1.92 ± 1.02	0.135^∧^
Total cholesterol (mmol/L)	5.24 ± 4.65	5.33 ± 2.80	0.747^∧^	4.65 ± 1.28	0.133^∧^	4.74 ± 1.28	0.489^∧^
LDL (mmol/L)	2.89 ± 0.96	3.12 ± 1.52	0.531^∧^	2.64 ± 1.01	0.065^∧^	2.77 ± 0.94	0.999^∧^
HDL (mmol/L)	1.12 ± 0.28	1.16 ± 0.33	0.219^∧^	1.18 ± 0.70	0.254^∧^	1.08 ± 0.35	0.294^∧^
Fasting blood glucose (mmol/L)	9.46 ± 4.19	9.87 ± 4.73	0.999^∧^	8.99 ± 5.92	0.287^∧^	8.41 ± 4.03	0.155^∧^
Glutamic-pyruvic transaminase (IU/L)	34.71 ± 53.69	**25.90** **±** **20.00**	0.021^*^^∧^	**24.23** **±** **24.53**	0.005^*^^∧^	**20.45** **±** **14.56**	<0.001^**^^∧^
Serum creatinine (umol/L)	66.20 ± 33.66	**100.45 ±** **126.52**	0.001^*^^∧^	**79.07**± **32.44**	<0.001^**^^∧^	**103.08**± **72.53**	<0.001^**^^∧^
Blood uric acid (umol/L)	348.00 ± 114.23	371.61 ± 134.08	0.591^∧^	364.83 ± 113.47	0.753^∧^	**382.94**± **108.93**	0.013^*^^∧^
Post-prandial blood glucose (mmol/L)	15.60 ± 5.90	16.06 ± 5.98	0.435^∧^	15.46 ± 5.63	0.838^∧^	15.01 ± 4.77	0.347^∧^
HbA1c (%)	8.95 ± 2.53	9.16 ± 2.63	0.420^∧^	8.56 ± 2.20	0.194^∧^	8.58 ± 2.33	0.250^∧^
Fasting C-peptide (ng/ml)	1.58 ± 1.38	1.41 ± 1.08	0.360^∧^	1.61 ± 1.23	0.991^∧^	1.51 ± 1.20	0.990^∧^
Post-prandial 1 h C-peptide (ng/ml)	3.30 ± 3.88	2.93 ± 2.59	0.658^∧^	3.04 ± 2.24	0.577^∧^	2.86 ± 2.20	0.697^∧^
Post-prandial 2 h C-peptide (ng/ml)	4.22 ± 5.18	3.58 ± 3.27	0.117^∧^	4.22 ± 3.68	0.979^∧^	3.95 ± 2.81	0.678^∧^
eGFR (mL/min)	85.72 ± 17.97	**73.16** **±** **21.26**	<0.001^**^^∧^	**73.19** **±** **19.51**	<0.001^**^^∧^	**64.59** **±** **21.56**	<0.001^**^^∧^
Duration of diabetes (years)	5.55 ± 5.45	**7.86** **±** **6.54**	0.001^*^^∧^	**9.44** **±** **6.50**	<0.001^**^^∧^	**10.99** **±** **6.78**	<0.001^**^^∧^
Family history of diabetes [*n*(%)]	56 (21.05%)	54 (19.49%)	0.467^Δ^	18 (16.67%)	0.335^Δ^	18 (18.75%)	0.695^Δ^
Former smoking [*n*(%)]	10 (3.76%)	5 (2.20%)	0.330^Δ^	**10 (9.26%)**	0.045^*^^Δ^	4 (4.17%)	0.865^Δ^
Current smoking [*n*(%)]	43 (21.13%)	37 (23.79%)	0.968^Δ^	17 (21.13%)	0.919	15 (15.1%)	0.9015
Current drinking [*n*(%)]	23 (8.65%)	18 (6.50%)	0.774^Δ^	8 (7.41%)	0.694	10 (10.42%)	0.606
Patients with Hypertension [*n*(%)]	40 (15.04%)	**78 (28.16%)**	<0.001^**^^Δ^	**75 (69.44%)**	<0.001^**^^Δ^	**68 (70.83%)**	<0.001^**^^Δ^

a vs. T2DM patients without any complication;

* *P <0.05*,

** *P <0.001*.

Δ the P-value of χ^2^-test and Bonferroni correction;

∧ *the P-values of Kruskal-Wallis test and Bonferroni correction*.

*BMI, body mass index; LDL, low density lipoprotein; HDL, high density lipoprotein; HbA1c, glycated hemoglobin; eGFR, estimated glomerular filtration rate*.

*The bold in the table means statistically significant (P <0.05)*.

*XPO5* rs11077, *TARBP2* rs784567, *DICER1* rs13078, and rs3742330 met the requirement of the HWE in healthy controls. However, *RAN* rs14035 deviated from HWE in healthy controls (Tables S2, S3).

### Association Between T2DM and *XPO5*/*DICER1*/*TARBP2* Polymorphisms

We identified the effects of *XPO5, DICER1*, and *TARBP2* SNPs on T2DM. As presented in Table 2, there was a significant association between rs13078 in the *DICER1* gene and a decreased risk of developing T2DM under the allelic mode (A vs. T: OR: 0.64; 95CI%: 0.42–0.95; P: 0.026), suggesting that individuals carrying the A allele had a 36% lower risk of developing T2DM than those carrying the T allele. However, there were no significant differences between T2DM under the allelic and codominant, dominant, recessive models, and other SNPs.

### Association Between T2DM Vascular Complication and *RAN*/*XPO5*/*DICER1*/*TARBP2* Polymorphisms

The effects of *RAN, XPO5, DICER1*, and *TARBP2* SNPs on diabetes progression were further analyzed. We found that rs14035 in the *RAN* gene was associated with T2DM with macrovascular complications (Tables 3, 4). In comparison with T2DM patients with the CC genotype, T2DM patients carrying TT+CT genotypes at rs14035 had 1.89-fold higher risk of suffering T2DM macrovascular complications (TT+CT vs. CC: OR: 1.89; 95%CI: 1.04–3.45; *P*: 0.037). However, we did not find that the rest of target SNPs in *XPO5, DICER1*, and *TARBP2* genes were associated with the susceptibility of T2DM vascular complications.

**Table 3:** Association of *XPO5, DICER1*, and *TARBP2* polymorphisms with T2DM.

**Gene**	**SNP**	**Model**	**Genotype**	**Case**	**Control**	**OR (95%CI)**	***P*-value**
*XPO5*	rs11077	Codominant^a^	TT	643	455	1.00 (Ref)	
			GG	0	1	NA	NA
			TG	100	76	0.88 (0.59–1.32)	0.876
		Dominant^a^	GG+TG	100	77	0.88 (0.59–1.31)	0.521
		Recessive^a^	TT+TG	743	531	1.00 (Ref)	
			GG	0	1	NA	NA
		Allelic^b^	T	1386	986	1.00 (Ref)	
			G	100	78	0.91 (0.67–1.24)	0.557
*DICER1*	rs13078	Codominant^a^	TT	698	480	1.00 (Ref)	
			AA	2	0	NA	NA
			TA	43	52	0.61 (0.36–1.03)	0.064
		Dominant^a^	AA+TA	47	52	0.66 (0.39–1.11)	0.115
		Recessive^a^	TT+TA	741	532	1.00 (Ref)	
			AA	2	0	NA	NA
		Allelic^b^	T	1439	1012	1.00 (Ref)	
			A	47	52	**0.64 (0.42–0.95)**	0.026^*^
*DICER1*	rs3742330	Codominant^a^	AA	323	209	1.00 (Ref)	
			GG	92	60	1.02 (0.65–1.64)	0.910
			AG	328	263	0.86 (0.64–1.16)	0.330
		Dominant^a^	GG+AG	420	323	0.89 (0.67–1.19)	0.433
		Recessive^a^	AA+AG	651	472	1.00 (Ref)	
			GG	92	60	1.11 (0.72–1.72)	0.633
		Allelic**^b^**	A	974	681	1.00 (Ref)	
			G	512	383	0.93 (0.79–1.10)	0.421
TARBP2	rs784567	Codominant^a^	GG	734	500	1.00 (Ref)	
			AA	0	0	NA	NA
			GA	8	12	0.53 (0.19–1.45)	0.213
		Dominant^a^	GA+AA	8	12	0.53 (0.19–1.45)	0.213
		Allelic^b^	G	1476	1012	1.00 (Ref)	
			A	8	12	0.46 (0.19–1.12)	0.080

* *P <0.05*.

a *Adjusting BMI, body mass index; LDL, low density lipoprotein; HDL, high density lipoprotein; and TG, triglyceride*.

b *OR, P-value were from χ^2^-tests and adjusted no variable*.

*The bold in the table means statistically significant (P <0.05)*.

**Table 4:** Association of *RAN, XPO5, DICER1*, and *TARBP2* polymorphisms with vascular complications of T2DM.

**Gene**	**SNP**	**Model**	**Genotype**	**Microvascular complications vs. T2DM alone**		**Macrovascular complications vs. T2DM alone**		**Micro-macrovascular complications vs. T2DM alone**	
				**OR (95CI%)**	***P*-value**	**OR (95CI%)**	***P*-value**	**OR (95CI%)**	***P*-value**
*RAN*	rs14035	Codominant^a^	CC	1.00 (Ref)		1.00 (Ref)		1.00 (Ref)	
			TT	0.8 (0.32–2.04)	0.635	2.24 (0.7–7.21)	0.177	0.81 (0.15–4.42)	0.800
			CT	1.3 (0.83–2.02)	0.258	1.83 (0.97–3.46)	0.066	1.61 (0.85–3.06)	0.152
		Dominant^a^	TT+CT	1.21 (0.8–1.83)	0.390	**1.89 (1.04–3.45)**	0.037^*^	1.5 (0.81–2.78)	0.209
		Recessive^a^	CC+CT	1.00 (Ref)		1.00 (Ref)		1.00 (Ref)	
			TT	0.75 (0.3–1.89)	0.533	1.86 (0.59–5.82)	0.292	0.7 (0.13–3.79)	0.674
		Allelic^b^	C	1.00 (Ref)		1.00 (Ref)		1.00 (Ref)	
			T	1.07 (0.77–1.48)	0.689	1.33 (0.90–1.96)	0.157	1.08 (0.71–1.66)	0.710
*XPO5*	rs11077	Codominant^a^	TT	1.00 (Ref)		1.00 (Ref)		1.00 (Ref)	
			TG	1.32 (0.77–2.27)	0.318	0.62 (0.25–1.52)	0.292	0.99 (0.4–2.46)	0.968
		Dominant^a^	GG+TG	1.32 (0.77–2.27)	0.318	0.62 (0.25–1.52)	0.292	0.99 (0.4–2.46)	0.968
		Allelic^b^	T	1.00 (Ref)		1.00 (Ref)		1.00 (Ref)	
			G	1.26 (0.78–2.04)	0.345	0.69 (0.34–1.42)	0.310	0.86 (0.43–1.74)	0.679
*DICER1*	rs13078	Codominant^a^	TT	1.00 (Ref)		1.00 (Ref)		1.00 (Ref)	
			TA	1.36 (0.61–3.01)	0.457	2.08 (0.6–7.16)	0.250	2.21 (0.63–7.81)	0.221
		Dominant^a^	AA+TA	1.19 (0.55–2.58)	0.662	1.78 (0.54–5.89)	0.349	2.21 (0.63–7.81)	0.221
		Allelic^b^	T	1.00 (Ref)		1.00 (Ref)		1.00 (Ref)	
			A	0.92 (0.466–1.84)	0.819	0.64 (0.24–1.74)	0.377	0.72 (0.27–1.96)	0.521
*DICER1*	rs3742330	Codominant^a^	AA	1.00 (Ref)	–	1.00 (Ref)	–	1.00 (Ref)	–
			GG	0.99 (0.55–1.81)	0.973	0.63 (0.25–1.55)	0.308	0.65 (0.27–1.62)	0.352
			AG	0.85 (0.56–1.29)	0.425	0.88 (0.48–1.6)	0.654	0.71 (0.38–1.32)	0.269
		Dominant^a^	GG+AG	0.88 (0.6–1.3)	0.512	0.81 (0.46–1.43)	0.461	0.69 (0.39–1.24)	0.214
		Recessive^a^	AA+AG	1.09 (0.62–1.89)	0.787	0.67 (0.29–1.58)	0.356	0.78 (0.33–1.83)	0.555
			GG	1.00 (Ref)	–	1.00 (Ref)	–	1.00 (Ref)	–
		Allelic^b^	A	1.00 (Ref)	–	1.00 (Ref)	–	1.00 (Ref)	–
			G	1.02 (0.78–1.32)	0.894	0.83 (0.59–1.16)	0.270	0.86 (0.80–1.22)	0.394
*TARBP2*	rs784567	Codominant^a^	GG	1.00 (Ref)		1.00 (Ref)		1.00 (Ref)	
			AA	1.19 (0.55–2.58)	0.662	1.95 (0.09–44.24)	0.678	NA	–
		Dominant^a^	GA+AA	1.19 (0.55–2.58)	0.662	1.95 (0.09–44.24)	0.678	NA	–
		Allelic^b^	G	1.00 (Ref)		1.00 (Ref)		1.00 (Ref)	
			A	1.57 (0.32–10.75)	0.703	0.82 (0.02–10.2)	0.703	NA	NA

* *P <0.05*.

a *Adjusting age, duration of type 2 diabetes and hypertension*.

b *P-value were from χ^2^-tests and adjusted no variable*.

*The bold in the table means statistically significant (P <0.05)*.

### Association Among T2DM, T2DM Vascular Complications, Gene-Environment, and Gene-Gene Interaction

**Figure 2:**
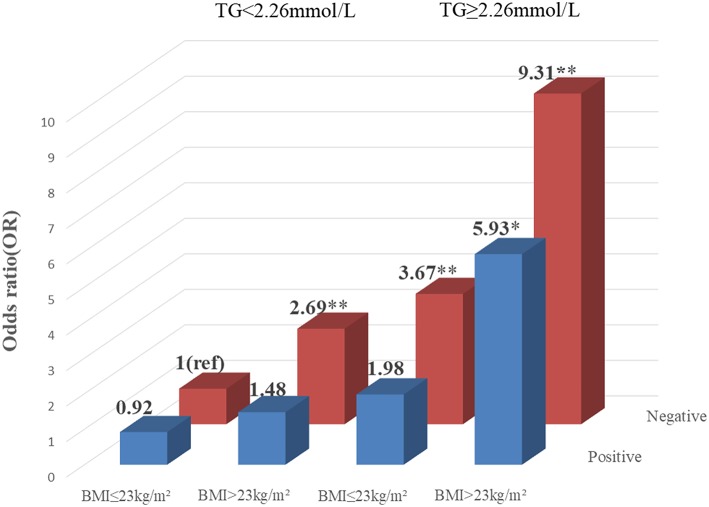
Risk analysis of gene-environment interaction Model: *DICER1* rs13078, BMI, TG. The reference group is the interaction of wild-type for rs13078, normal BMI and normal TG. The OR value is presented in the figure. ^*^P < 0.05 and ^**^P < 0.001.

**Figure 3:**
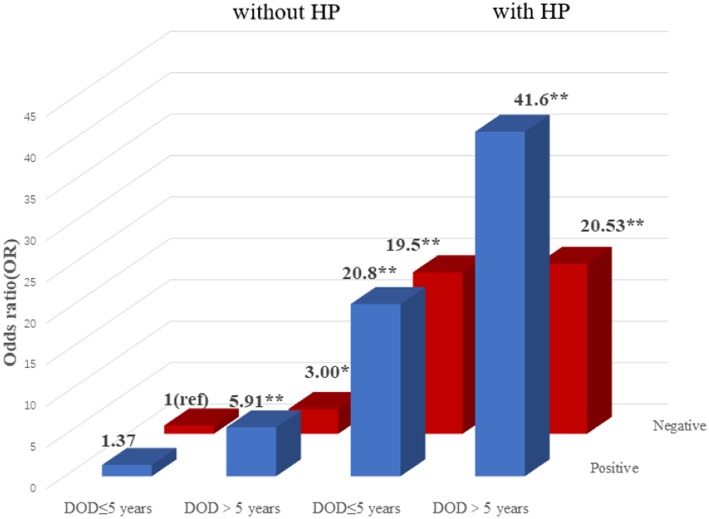
Risk analysis of gene-environment interaction for T2DM with macrovascular complications Model: *RAN* rs14035, DOD, HP. The reference group is the interaction of wild-type for rs14035, duration of T2DM <5 years and without hypertension. The OR value is shown in the figure. ^*^P < 0.05 and ^**^P < 0.001.

To explore the association among gene-environment interaction, T2DM and its vascular complications, MDR analysis was conducted to analysis the five SNPs in the *RAN, XPO5, DICER1*, and *TARBP2* genes as well as environment factors (Table S4). The best gene-environmental interaction model on T2DM and its macrovascular complications were identified (*DICER1* rs13078, BMI and TG; *RAN* rs14035, DOD and HP), with a significant TBA value and the highest CVC value (Table 5). The risk analysis of the three-way interaction in the model was also performed (Table S5). As shown in Figure 2, in comparison with the reference group (wild-type rs13078, normal BMI, and normal TG), the individuals with all three factors (mutation of rs13078, high BMI, and high TG) exhibited a 5.93-fold higher possibility of developing T2DM (OR: 5.93; 95%CI:1.25–28.26; *P*: 0.025). Figure 3 shows that the individuals with mutation of *RAN* rs14035, DOD more than 5 years and hypertension had a 40.60-fold higher possibility of suffering diabetic macrovascular complications when compared with the reference group (wild-type RAN rs14035, DOD <5 years and without hypertension) (OR: 41.60; 95%CI:11.75–147.35; *P* < 0.001). However, no other gene-environment interactions were identified for T2DM and T2DM vascular complications.

**Table 5:** The gene-environment interaction model using MDR analysis for T2DM and its vascular complications.

**Group**	**Model**	**CVC**	**TBA**	***P*-value**
T2DM vs. Healthy controls	rs13078^*^BMI^*^TG	10/10	0.6496	0.014^*^
T2DM with macrovascular complication vs. T2DM alone	rs14035^*^HP^*^DOD	10/10	0.7771	0.025^*^

* *P <0.05*.

*CVC, cross validation consistency; TBA, testing balance accuracy; BMI, body mass index; TG, triglyceride*.

However, no gene-gene interaction was found to be associated with the increase risk of T2DM and its vascular complications using MDR analysis.

### Association Among T2DM, Its Vascular Complications, and *DICER1* Haplotype Frequencies

The two SNPs (rs3742330 and rs13078) constitute a haplotype block spanning 3 kb of the *DICER1* gene (Figure 3). We conducted a haplotype analysis among the *DICER1* gene, T2DM and T2DM vascular complications. As shown in Table S6, compared with the highest frequency haplotype AT, the haplotype AA was significantly related to a lower risk of T2DM (OR: 0.63; 95%CI: 0.42–0.94; *P*: 0.023).

## Discussion

Alterations in the miRNA machinery play important roles in the pathogenesis of a variety of disorders (49), which may account for abnormal profiles of miRNA in various diseases. Recently, more and more studies have provided evidence that alterations in miRNA machinery may result in dysregulation or upregulation of miRNAs in diabetes (40, 50, 51). In our study, a significant 0.64-fold decrease was found in the A allele frequency at *DICER1* rs13078 in T2DM patients than healthy individuals in the allelic model, suggesting that individuals carring the A allele at *DICER1* rs13078 had a decreased possibility of developing T2DM than those carrying the T allele. Additionally, we also identified a protective effect of the AA haplotype in DICER1.

*DICER1*, which situate in chromosome 14q32.13, contains 1922 amino acids in humans, encoding an approximately 218 kDa RNase III endonuclease (21, 52). The DICER1 enzyme is responsible for the processing of gene-encoded pre-miRNAs into mature miRNAs, and it plays a key role in the highly conserved cellular pathway (52). *DICER1* is also well known to be an important component in the oncogenic process of several cancers, such as breast cancer (53), hepatocellular carcinoma (54), lung cancer (55), and ovarian cancer (56). In metabolic diseases, Noren Hooten et al. (57) hypothesized that alterations of the levels of *DICER1* gene may play an important role in organismal aging and the upregulation of expression of *DICER1* gene may provide us a new pharmacotherapeutic approaches for age-related disease, such as T2DM. Furthermore, it has been demonstrated that different levels of expression of microRNAs were identified in exosomes isolated from the serum and in the blood of healthy control individuals compared with patients with T2DM, hypercholesterolemia or metabolic syndrome (58, 59). It has been reported that the site of *DICER1* rs13078 located in the 3′-UTR of the *DICER1* gene and might be an important component in the expression process of the *DICER1* gene by influencing the miRNAs binding site, which might impact the DICER1 enzyme's function by *DICER1* gene regulation and sequentially impact the expression of microRNAs (52, 60, 61). Additionally, rs13078 genetic variants in *DICER1* have been reported to be related to other diseases, such as gestational hypertension (62) and larynx cancer (63).

In the association analysis between diabetic vascular complications and SNPs, we found that T2DM patients who carried TT+TC genotype of rs14035 in *RAN* gene had a 1.89-fold increased risk of developing macrovascular complications compared with those with the CC wild-type genotype. *RAN* rs14035 is also located in the 3′-UTR of the *RAN* gene at chr12:130876696 (64). The RAN enzyme as the member of the Ras superfamily of GTPases, plays a key component in the translocation of pre-miRNAs from the nucleus to the cytoplasm through the nuclear pore complex (65). RAN-GTP is depleted as a result of RAN guanine nucleotide exchange factor inhibition, and pre-miRNA export is greatly reduced, indicating that miRNA transport is mediated by a RAN-GTP-binding export receptor (66). These data not only suggest that mutations in *RAN* might play an important role in pathology-related changes in the transport and expression of miRNA but also imply that polymorphisms of the *RAN* gene might also be related to the biosynthesis and translation of miRNAs. Vorpahl et al. (67) suggested that inhibition or modulation of RAN-GTPase-activating protein expression might be sufficient to attenuate the vascular proliferative response following vascular injury. Additionally, RAN-GTPase signaling might also be essential for postnatal pancreatic islet development and glucose homeostasis (68). It has been reported that a significant upregulation of circulating miR-126 has been detected in patients with angina and acute myocardial infarction, while downregulation of miR-126 has been detected in plasma from patients with cancer, diabetes or heart failure (58, 59). Moreover, it has been reported that circulating miR-126-3p, which may be a reliable biomarker of physiological endothelial senescence in elderly subjects with normoglycemia, underlies a mechanism that may be disrupted in elderly patients with DM (69). These studies give evidence the rs14035 polymorphism in *RAN* might be related to the occurrence and deterioration of the macrovascular complications of T2DM by regulating the expression level of *RAN*.

Recently, it has been reported that the susceptibility of type 2 diabetes mellitus and vascular complications of T2DM could be affected by various factors, including interactions between various environmental factors and the different polymorphisms (10). An MDR analysis was conducted to further investigate the multiple-factor, higher-order interaction of T2DM and the vascular complications of T2DM. The best three-factor interaction model among *DICER1* rs13078, BMI (body mass index) and TG (triglyceride), suggested that rs13078 in *DICER1* more likely interacts with overweigh and high TG to increase the possibility of developing T2DM. This outcome is consistent with the analysis of the clinical data between healthy control individuals and T2DM patients which showed that T2DM patients had significantly higher BMI and TG than healthy controls. Shai et al. (70) and Astrup et al. (71) also supported that individuals with abnormal lipid metabolism (high TG, TC, HDL, and low HDL) had a higher risk of developing T2DM. It has also been reported that BMI above normal weight level has been demonstrated to be one of the risk factors of T2DM and its complications (71–73). Moreover, our interaction results were supported by the synergistic effect and individual effect that overweight, increased triglyceride, high LDL and low HDL are associated with T2DM risk (74). In addition, another best three-factor interaction model (*RAN* rs14035, with hypertension and more than 5 years of T2DM duration) was also identified to be related to the increased risk of macrovascular complications of T2DM. Hypertension is known as one of the independent risk factors of T2DM (75). Moreover, the coexistence of hypertension and diabetes will significantly increase the risk of developing cardiovascular complications of DM, which means that hypertension may be an important risk factor for diabetic macrovascular complications (76, 77). According to the ADA guidelines, with the increase in the duration of diabetes, the risk of developing diabetic vascular complications will significantly increase (78). Many studies also supported that the duration of diabetes played an important role in developing diabetic vascular complications (79, 80). However, our study is the first time we found these two interaction models in T2DM and diabetic macrovascular complications, thus further studies with larger population samples are needed.

Similarly, there might be more environmental factors, such as diet behavior, passive smoke exposure and PM_2.5_, which might also modify the effects of genetic variants on the susceptibility of T2DM and its vascular complications (72, 73). For example, the study by Eze et al. demonstrated the interaction between long-term PM_10_ and gene polymorphisms at IL6-572 on T2DM (81). Therefore, more environmental factors could be included in further studies on interaction.

Our study has several limitations. First, the sample in the study was not very large, which might restrict the ability to explore weaker associations among T2DM, vascular complications of T2DM and SNPs. Second, the basic characteristics of the included individuals collected were not very comprehensive. Several clinical characteristics were not collected, such as the actual values of systolic and diastolic blood pressure. In addition, we should also include more meaningful environmental factors, such as diet, PM_10_ and passive smoke, in our analysis. Therefore, more large and well-designed investigation is needed to support our findings.

As far as we know, this is the first study to investigate the association of these five polymorphisms (rs14035 in *RAN*, rs11077 in *XPO5*, rs13078 in *DICER1*, rs3742330 in *DICER1* and rs784567 in *TARBP2*) with the risk of T2DM and its vascular complications. Our findings offer us the first evidence that rs13078 in *DICER1* is related to a reduced risk of T2DM and that *RAN* rs14035 is relevant to an increased risk of macrovascular complication of T2DM in southern Chinese population. Additionally, our results also demonstrate two feasible interactions, *DICER1* rs13078, BMI and TG in T2DM and *RAN* rs14035, hypertension and duration of T2DM in diabetic macrovascular complications. Our study may provide a new clue of epidemiology about the importance of miRNA processing genes (*RAN, XPO5, DICER1*, and *TARBP2*) in type 2 diabetes mellitus and diabetic vascular complications.

## Ethics Statement

Our study was conducted in conformity with the rules of the Ethics Committee of Jinan University by written informed consent to all participant subjects. All subjects gave written informed consent in accordance with the Declaration of Helsinki. The Ethics Committee of Jinan University approved the protocol.

## Author Contributions

ZW, XZ, and XX contributed equally to the writing of this paper. LN provided all the samples for this study. SZ, XC, KZ, SD, JL, XH, DL, YW, JW, YL, YY, KL, CL, and BZ carried out data collection and the extraction of DNA. GY and CJ carried out whole design.

### Conflict of Interest Statement

The authors declare that the research was conducted in the absence of any commercial or financial relationships that could be construed as a potential conflict of interest.
