# Supplementary material for: Association of Polymorphisms in miRNA Processing Genes With Type 2 Diabetes Mellitus and Its Vascular Complications in a Southern Chinese Population
Source: Front Endocrinol (Lausanne). 2019 Jul 12;10:461. doi: 10.3389/fendo.2019.00461 (PMC6639830; doi:10.3389/fendo.2019.00461)
Supplement: Supplementary file 1 [file Table_1.DOCX]

**Supplementary table**

| Gene | SNP | Location | Alleles | MAF (%) case/control | HWE | Call rate (%) |
| --- | --- | --- | --- | --- | --- | --- |
| RAN | rs14035 | 3’-UTR | C/T | 19.43/18.50 | **<0.001*** | 99.37 |
| XPO5 | rs11077 | 3’-UTR | T/G | 6.73/7.33 | 0.235 | 99.92 |
| DICER1 | rs3742330 | 3’-UTR | A/G | 34.45/36.00 | 0.093 | 99.92 |
|  | rs13078 | 3’-UTR | T/A | 3.16/4.89 | 0.236 | 99.92 |
| TARBP2 | rs784567 | Promoter | G/A | 0.54/1.17 | 0.792 | 99.84 |

**Table S.1** General information for *RAN, XPO5, DICER1* and *TARBP2* polymorphisms.

SNP, single nucleotide polymorphism; MAF, minor allele frequency; HWE, Hardy-Weinberg equilibrium; UTR, untranslated region.

*HWE<0.05.

**Table S.2** The Kolmogorov-Smirnov normality test of quantitative variables in healthy control group and T2DM group

| Characteristics | healthy control group  (N = 532) | | | | T2DM group  (N = 743) | | | |
| --- | --- | --- | --- | --- | --- | --- | --- | --- |
|  |  | statistic | P value |  |  | statistic | P value |  |
| Age (years) | **0.051** | | **0.002*** | | **0.056** | | **<0.001**** | |
| BMI (kg/m^2^) | **0.049** | | **0.008*** | | **0.048** | | **0.001*** | |
| TG (mmol/L) | **0.143** | | **<0.001**** | | **0.188** | | **<0.001**** | |
| HDL cholesterol (mmol/L) | **0.077** | | **<0.001**** | | **0.146** | | **<0.001**** | |
| LDL cholesterol (mmol/L) | **0.043** | | **0.019*** | | **0.062** | | **<0.001**** | |
| Total cholesterol (mmol/L) | 0.038 | | 0.067 | | **0.228** | | **<0.001**** | |
| FBG (mmol/L) | **0.137** | | **<0.001**** | | **0.148** | | **<0.001**** | |
| Glutamic-pyruvic transaminase (IU/L) | **0.151** | | **<0.001**** | | **0.269** | | **<0.001**** | |
| Serum creatinine (umol/L) | **0.053** | | **0.001*** | | **0.267** | | **<0.001**** | |
| Blood uric acid (umol/L) | **0.064** | | **<0.001**** | | **0.058** | | **<0.001**** | |

^*^ *P* < 0.05, ^**^ *P* < 0.001; The bold in the table means statistically significant. (*P* < 0.05)

**Table S.3** The Kolmogorov-Smirnov normality test of quantitative variables in T2DM without complication group, T2DM with microvascular complications group, T2DM with macrovascular complications group and T2DM with microvascular-macrovascular complications group

| Characteristics | T2DM without complication group  (n=266) | | | | T2DM with microvascular complications group  (n=227) | | | | T2DM with macrovascular complications group  (n=108) | | | | T2DM with microvascular-  macrovascular complications group  (n=96) | | | |
| --- | --- | --- | --- | --- | --- | --- | --- | --- | --- | --- | --- | --- | --- | --- | --- | --- |
|  |  | statistic | P value |  |  | statistic | P value |  |  | statistic | P value |  |  | statistic | P value |  |
| Age (years) | **0.071** | | **0.003*** | | 0.046 | | 0.200 | | **0.095** | | **0.018*** | | 0.086 | | 0.074 | |
| BMI (kg/m^2^) | 0.046 | | 0.200 | | **0.078** | | **0.003*** | | **0.134** | | **<0.001**** | | 0.094 | | 0.083 | |
| TG (mmol/L) | **0.210** | | **<0.001**** | | **0.183** | | **<0.001**** | | **0.155** | | **<0.001**** | | **0.119** | | **0.002*** | |
| HDL cholesterol (mmol/L) | **0.099** | | **<0.001**** | | **0.126** | | **<0.001**** | | **0.263** | | **<0.001**** | | **0.168** | | **<0.001**** | |
| LDL cholesterol (mmol/L) | 0.040 | | 0.200 | | **0.135** | | **<0.001**** | | 0.085 | | 0.060 | | 0.063 | | 0.200 | |
| Total cholesterol (mmol/L) | **0.306** | | **<0.001**** | | **0.205** | | **<0.001**** | | 0.085 | | 0.061 | | 0.089 | | 0.064 | |
| FBG (mmol/L) | **0.146** | | **<0.001**** | | **0.132** | | **<0.001**** | | **0.228** | | **<0.001**** | | **0.171** | | **<0.001**** | |
| Glutamic-pyruvic transaminase (IU/L) | **0.305** | | **<0.001**** | | **0.194** | | **<0.001**** | | **0.245** | | **<0.001**** | | **0.173** | | **<0.001**** | |
| Serum creatinine (umol/L) | **0.195** | | **<0.001**** | | **0.315** | | **<0.001**** | | **0.136** | | **<0.001**** | | **0.231** | | **<0.001**** | |
| Blood uric acid (umol/L) | **0.074** | | **0.002*** | | **0.100** | | **<0.001**** | | 0.065 | | 0.200 | | 0.078 | | 0.187 | |
| Postprandial blood glucose (mmol/L) | **0.080** | | **0.001*** | | **0.086** | | **0.002*** | | 0.069 | | 0.200 | | 0.078 | | 0.200 | |
| HbA1c (%) | **0.097** | | **<0.001**** | | **0.093** | | **<0.001**** | | **0.121** | | **0.002*** | | **0.151** | | **<0.001**** | |
| Fasting C-peptide (ng/ml) | **0.181** | | **<0.001**** | | **0.156** | | **<0.001**** | | **0.170** | | **<0.001**** | | **0.153** | | **<0.001**** | |
| Postprandial 1hour C-peptide (ng/ml) | **0.229** | | **<0.001**** | | **0.157** | | **<0.001**** | | 0.129 | | 0.085 | | 0.163 | | 0.005* | |
| Postprandial 2hour C-peptide (ng/ml) | **0.222** | | **<0.001**** | | **0.149** | | **<0.001**** | | **0.151** | | **<0.001**** | | **0.151** | | **<0.001**** | |
| eGFR (mL/min) | **0.078** | | **0.030*** | | **0.079** | | **0.009*** | | 0.088 | | 0.200 | | 0.064 | | 0.200 | |
| Duration of diabetes (years) | **0.172** | | **<0.001**** | | **0.135** | | **<0.001**** | | **0.153** | | **<0.001**** | | **0.239** | | **<0.001**** | |

^*^ *P* < 0.05, ^**^ *P* < 0.001; The bold in the table means statistically significant. (*P* < 0.05)

**Table S.4** Summary of the MDR interaction models in gene-gene interaction

| Group | Model | TBA | CVC | P |
| --- | --- | --- | --- | --- |
| T2DM vs. Healthy controls | rs3742330 | 0.5011 | 8/10 | 0.172 |
|  | rs13078*rs3742330 | 0.5061 | 8/10 | 0.377 |
|  | rs11077*rs13078*rs784567 | 0.4891 | 4/10 | 0.623 |
|  | rs11077*rs13078*rs3742330*rs784567 | 0.4985 | 10/10 | 0.623 |
| T2DM with Micro vs. T2DM alone | rs14035 | 0.4823 | 5/10 | 0.507 |
|  | rs11077*rs3742330 | 0.4558 | 6/10 | 0.489 |
|  | rs11077*rs14035*rs3742330 | 0.4729 | 5/10 | 0.504 |
|  | rs11077*rs13078*rs14035*rs3742330 | 0.4590 | 7/10 | 0.518 |
| T2DM with Macro vs. T2DM alone | rs14035 | 0.4985 | 7/10 | 0.343 |
|  | rs14035*rs3742330 | 0.5366 | 9/10 | 0.370 |
|  | rs11077*rs14035*rs3742330 | 0.5280 | 5/10 | 0.365 |
|  | rs11077*rs13078*rs14035*rs3742330 | 0.5535 | 10/10 | 0.308 |
| T2DM with Micro-macro vs. T2DM alone | rs3742330 | 0.5017 | 8/10 | 0.623 |
|  | rs11077*rs3742330 | 0.4878 | 7/10 | 0.623 |
|  | rs11077*rs14035*rs3742330 | 0.5193 | 5/10 | 0.623 |
|  | rs11077*rs13078*rs14035*rs3742330 | 0.5396 | 9/10 | 0.172 |

TBA, testing balanced accuracy; CVC, cross validation consistency.

**Table S.5** Summary of the MDR interaction models in gene-environment interaction

| Group | Model | TBA | CVC | P |
| --- | --- | --- | --- | --- |
| T2DM vs. Healthy controls | BMI | 0.6259 | 10/10 | 0.035* |
|  | BMI*TG | 0.6431 | 10/10 | 0.017* |
|  | rs13078*BMI*TG | 0.6496 | 10/10 | 0.014* |
|  | BMI*TC*TG*HDL | 0.6278 | 7/10 | 0.030* |
| T2DM with Micro vs. T2DM alone | HP | 0.5971 | 10/10 | 0.172 |
|  | HP*DOD | 0.5723 | 7/10 | 0.293 |
|  | rs14035*HP*TC | 0.5128 | 2/10 | 0.647 |
|  | BMI*HP*DOD*TG | 0.5742 | 8/10 | 0.281 |
| T2DM with Macro vs. T2DM alone | HP | 0.7720 | 10/10 | 0.022* |
|  | rs14035*HP | 0.7720 | 10/10 | 0.022* |
|  | rs14035*HP*DOD | 0.7771 | 10/10 | 0.025* |
| T2DM with Micro-macro vs. T2DM alone | HP | 0.7793 | 10/10 | 0.024* |
|  | rs784567*HP | 0.7661 | 6/10 | 0.030* |
|  | HP*TG*HDL | 0.7456 | 4/10 | 0.049* |
|  | BMI*HP*DOD*TG | 0.7526 | 4/10 | 0.042* |

T2DM, type 2 diabetes, Micro, microvascular complication; Macro, macrovascular; Micro-macro, microvascular and macrovascular complication; BMI, body mass index; TG, triglyceride; TC, total cholesterol; HP, hypertension; DOD, duration of diabetes; TBA, testing balanced accuracy; CVC, cross validation consistency.

* P < 0.05.

**Table S.6** The relation of haplotypes in DICER1 genes with T2DM and T2DM vascular complications

| Haplotype | T2DM vs. Healthy controls | |  | T2DM with Micro vs.  T2DM alone | |  | T2DM with Macro vs.  T2DM alone | |  | T2DM with Micro-macro vs.  T2DM alone | |
| --- | --- | --- | --- | --- | --- | --- | --- | --- | --- | --- | --- |
|  | OR (95%CI) | P |  | OR (95%CI) | P |  | OR (95%CI) | P |  | OR (95%CI) | P |
| DICER1^a^ |  |  |  |  |  |  |  |  |  |  |  |
| AT | 1(Ref) |  |  | 1(Ref) |  |  | 1(Ref) |  |  | 1(Ref) |  |
| GT | 0.91(0.77-1.07) | 0.251 |  | 1.01(0.78-1.32) | 0.919 |  | 1.26(0.90-1.78) | 0.177 |  | 0.84(0.59-1.20) | 0.344 |
| AA | **0.63(0.42-0.94)** | **0.023*** |  | 0.93(0.46-1.86) | 0.832 |  | 2.10(0.71-6.34) | 0.170 |  | 0.68(0.25-1.86) | 0.450 |
| GA | NA | NA |  | NA | NA |  | NA | NA |  | NA | NA |

T2DM, type 2 diabetes mellitus; OR, odds ratio; Micro, microvascular complications; Macro, macrovascular complications; Micro-macro, microvascular and macrovascular complications; NA, not available.

**^a^** DICER1 haplotype: rs3742330 and rs13078; * P<0.05.
